# Identification and Characterization of Organic Calcium Crystals in the *Plumeria pudica* and *Plumeria rubra* Latexes

**DOI:** 10.1021/acsomega.5c03229

**Published:** 2025-07-11

**Authors:** Larissa Barbosa Nogueira Freitas, Cleverson Diniz Teixeira de Freitas, Alejandro Pedro Ayala, Thiago Alves de Moura, Jefferson Soares de Oliveira, José Francisco de Carvalho Gonçalves, Márcio Viana Ramos

**Affiliations:** † Department of Biochemistry and Molecular Biology, 28121 Federal University of Ceará, Fortaleza, Ceará 60440-900, Brazil; ‡ Department of Physics, Federal University of Ceará, Fortaleza, Ceará 60455-760, Brazil; § Federal Institute of Education, Science and Technology of Ceará, Acaraú, Ceará 62580-000, Brazil; ∥ Department of Biomedicine, 603028 Federal University of the Parnaíba Delta, Parnaíba, Piauí 64202-020, Brazil; ⊥ Plant Physiology and Biochemistry Laboratory, 191073 National Institute of Amazonian Research - MCTI-INPA, Manaus 69060-001, Brazil

## Abstract

Latex is usually a milky fluid but can also be clear, brown, or orange. It consists of organic compounds (rubber particles and primary and secondary metabolites) and proteins dispersed in an aqueous phase. Four latex fluids were examined through optical microscopy, and organic crystals were found. Crystal structures were observed in *Plumeria rubra* and *P. pudica*, while no crystal was found in the latex of *Calotropis procera* and *Himatanthus drasticus*. Various sizes and shapes of crystals, including raphides, styloids, crystalline sands, and prismatic forms, were documented. The structures were stable for a 96 h period at 25 °C. The relative abundance of crystal forms varied based on whether the latex was crude or water-diluted. All crystal forms related to *P. pudica* were identified as hydrated calcium malonate [Ca­(C_3_H_2_O_4_)­(H_2_O)_2_]·2H_2_O, representing the first documentation of these structures in plants. Calcium malonate crystals also were identified in the latex of *P. rubra*, which were mainly in the forms of raphides and styloids. Nonetheless, the crystalline sands and prismatic forms found in *P. rubra* latex exhibited an additional carbon ring in their structure ([C_20_H_34_Ca_2_O_19_]) identified as cerberic acid B. This result also represents the first report of calcium-containing cerberic acid B. The results were discussed based on the possible functions of these molecules within laticifers.

## Graphical abstract

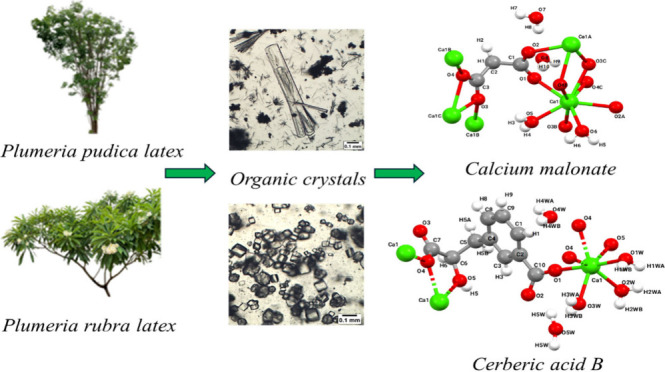

## Introduction

Laticifers are cells that exhibit morphological and metabolic differentiation and are present in various plant species across different taxonomic groups.^1^ Laticifers do not develop specialized tissues or organs. Instead, these cells elongate, forming distinct long rows that can further fuse and branch, creating autonomous networks within the tissues that are clearly distinguishable.^2^ These cells emerge during the initial stages of seed germination and plantlet development, and they maintain metabolic activity throughout the entire plant life cycle.^3^ There are many different types of biomolecules in laticifers, including rubber, secondary metabolites, lipids, and proteins.^4^ Furthermore, all cellular machinery essential for the basal metabolism of a plant cell exists within laticifers.^5^ This resulting fluid is referred to as latex.

Latex content varies among species and can also differ when comparing latex extracted from different parts of the same plant.^6^ This fluid is recognized for its capacity to store various secondary metabolites and proteins. Flavonoids, alkaloids, coumarins, steroids, saponins, polyphenols, and terpenoids are classes of secondary metabolites identified in latex,^4,7^ while the most common proteins are proteases, osmotins, lectins, chitin-binding proteins, and chitinases.^8^ These compounds have been associated with the plant’s defense against insects, herbivores, or pathogenic microorganisms.^6^ Conversely, no evidence for calcium crystals in latex has been found in the literature so far.

Calcium oxalate is the most prevalent crystal type in plants. The crystal shapes and sizes are diverse, and crystals are found in a variety of plant tissues and organs, such as bark, stems, fruits, flowers, leaves, roots, and seeds.^9,10^ Crystals are generally classified into five distinct classes based on their morphology, such as crystalline sand, raphide, druse, styloid, and prismatic.^11^ Studies indicate that the morphology of crystals produced by each plant species is consistent, indicating that they are genetically regulated and can be influenced by environmental factors such as light, temperature, and soil nutrients.^12^ The physiological functions that have been suggested for these crystals include detoxification, defense against herbivory, ion balance, and calcium regulation.^10^ It is intriguing that calcium oxalate crystals have not been described in latex fluids to date.

In general, the primary hypothesis suggests that organic calcium crystals are related to the sequestration or excretion of calcium ions (Ca^2+^) as well as the maintenance of ionic balance with a view to other minerals. This regulation of Ca crystals is crucial for plants because the calcium macronutrient acts as a structural element in cell walls and membranes. Additionally, it plays a more active role as an intracellular second messenger.^13^ Therefore, the uptake, distribution, and storage in the plant cell need to be tightly regulated.^14^ Plant crystals may function as a metabolic strategy for cells to preserve ionic balance and equilibrium for different cell activities.^12,15^ In this way, further research into the presence and function of these crystals in plant latex may reveal additional layers of defense strategies that plants employ in response to environmental or biotic stresses.^5^

The chemical composition of latex is mostly examined with samples that have undergone previous cleaning processes. Centrifugation, chemical precipitation, dialysis, and various other techniques aim to enhance accessibility to the targeted latex fraction.^16^ Nevertheless, these procedures may result in the depletion of components inherently present in a particular latex. This can be applied to organic crystals often described in plants but not in laticifers.^17^ We hypothesized that species belonging to the same botanical family can use different strategies to regulate the content of organic calcium crystals present in their latex fluids, which may also change qualitatively. Therefore, this study aimed to identify and characterize crystal forms of organic salts in the latex of four species from the Apocynaceae family (*Calotropis procera*, *Himatanthus drasticus*, *Plumeria pudica*, and *P. rubra*). Crystal structures were identified in only two Plumeria species and are the first records of calcium-containing crystals in plant latex.

## Results and Discussion

No crystal was found in the latex from *C. procera* and *H. drasticus* by optical microscopy, even after a time-course observation. As a result, these latex fluids were not investigated further. Conversely, *P. pudica* and *P. rubra* latex fluids, crude or diluted in water (1:3 ratio), exhibited distinct crystal formations (Figure 1). The crystals were observed shortly after the latex harvest. However, more precise methods would be required to confirm that the crystals are detected in the latex before collecting rather than generated subsequently. The crystals had a diverse array of sizes and shapes, such as raphides, styloids, crystalline sands, and prismatic. Although some crystals of the raphide type had a size of 200 μm × 10 μm, smaller raphides of around 50 μm long were more common. Larger in size, the styloids measured up to 910 × 120 μm. The diameters of the prismatic crystals and crystalline sand-type crystals were 310 μm × 250 and 18.7 μm × 20.6 μm, respectively (Figure 1).

**Figure 1:**
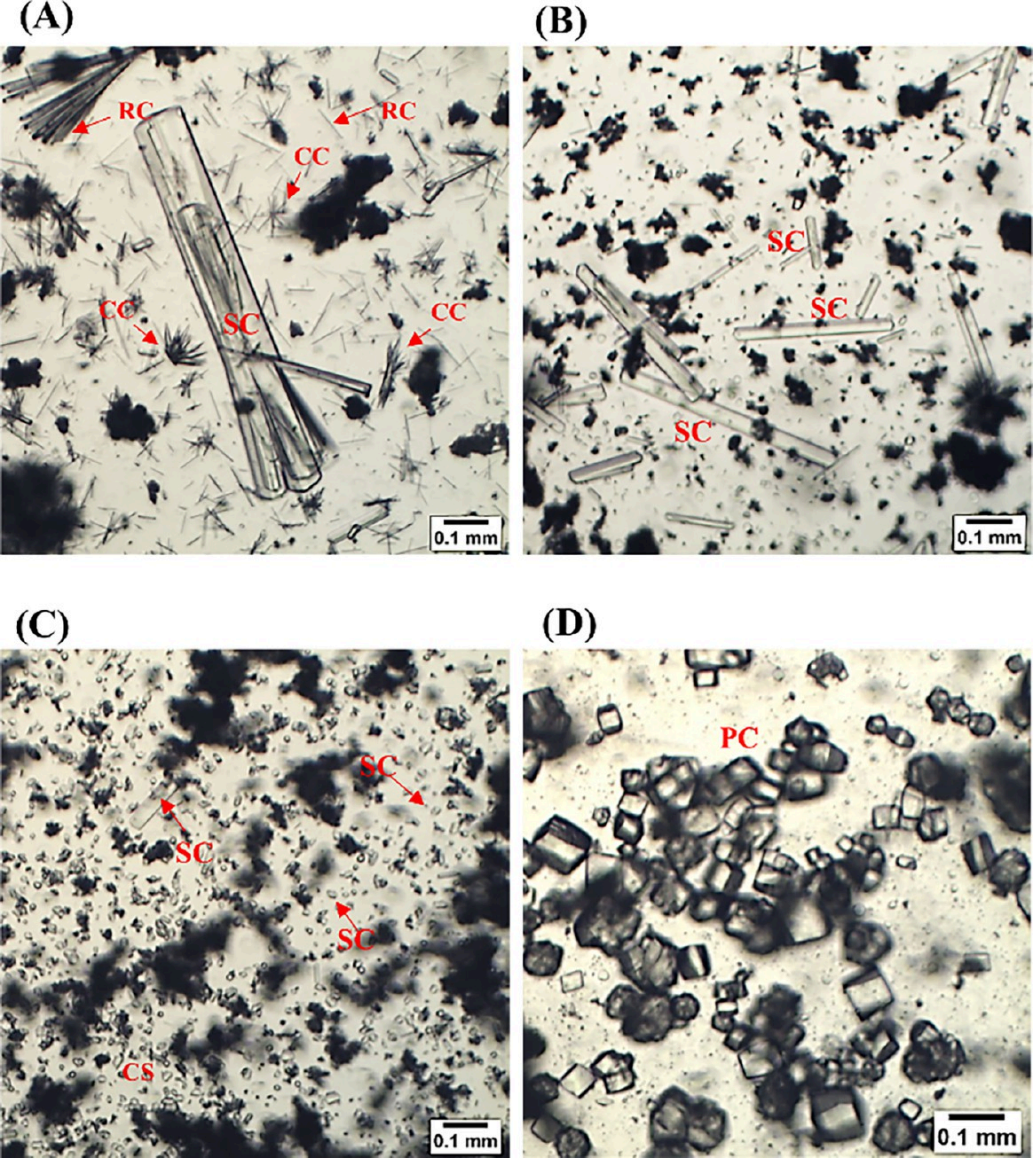
Morphology of crystals identified in the latex of *Plumeria pudica* and *P. rubra*. Images were obtained using a light microscope. A, B, and D: *P. pudica* latex. C: *P. rubra* latex. A, B, and C: samples diluted in distilled water (1:3 ratio). D: crude latex. Abbreviations: Raphid crystal (RC); styloid crystal (SC); crystalline sand (CS); prismatic crystal (PC); and aggregated crystal complex (CC).

Figures 2 and 3 indicate that there were no apparent changes in the crystal structures of the *P. rubra* and *P. pudica* latexes after 96 h at 25 °C. The time-course analysis revealed no alterations in the shape, size, or amount of the crystals. This observation indicates that crystals are not formed following latex collection. On the other hand, the crystals present in the crude latex of *P. pudica* displayed a distinct morphology compared to that of those derived from water-diluted latex. This was observed in only one sample (Pr2) of *P. rubra.* Prismatic and crystalline sand crystals were predominant in the crude latexes of both species, whereas complex aggregate crystals, raphids, and styloids were more prevalent in water-diluted latexes (Figures 2 and 3). These observed differences indicate that crystal formation may be influenced by the concentration of compounds in the medium as well as by the hydration conditions. In accordance, some studies show that the microenvironment in which crystallization occurs—the concentration of ions or other macromolecules in the solution—is crucial for controlling the nucleation, growth, and cessation of crystal formation.^10,11^

**Figure 2:**
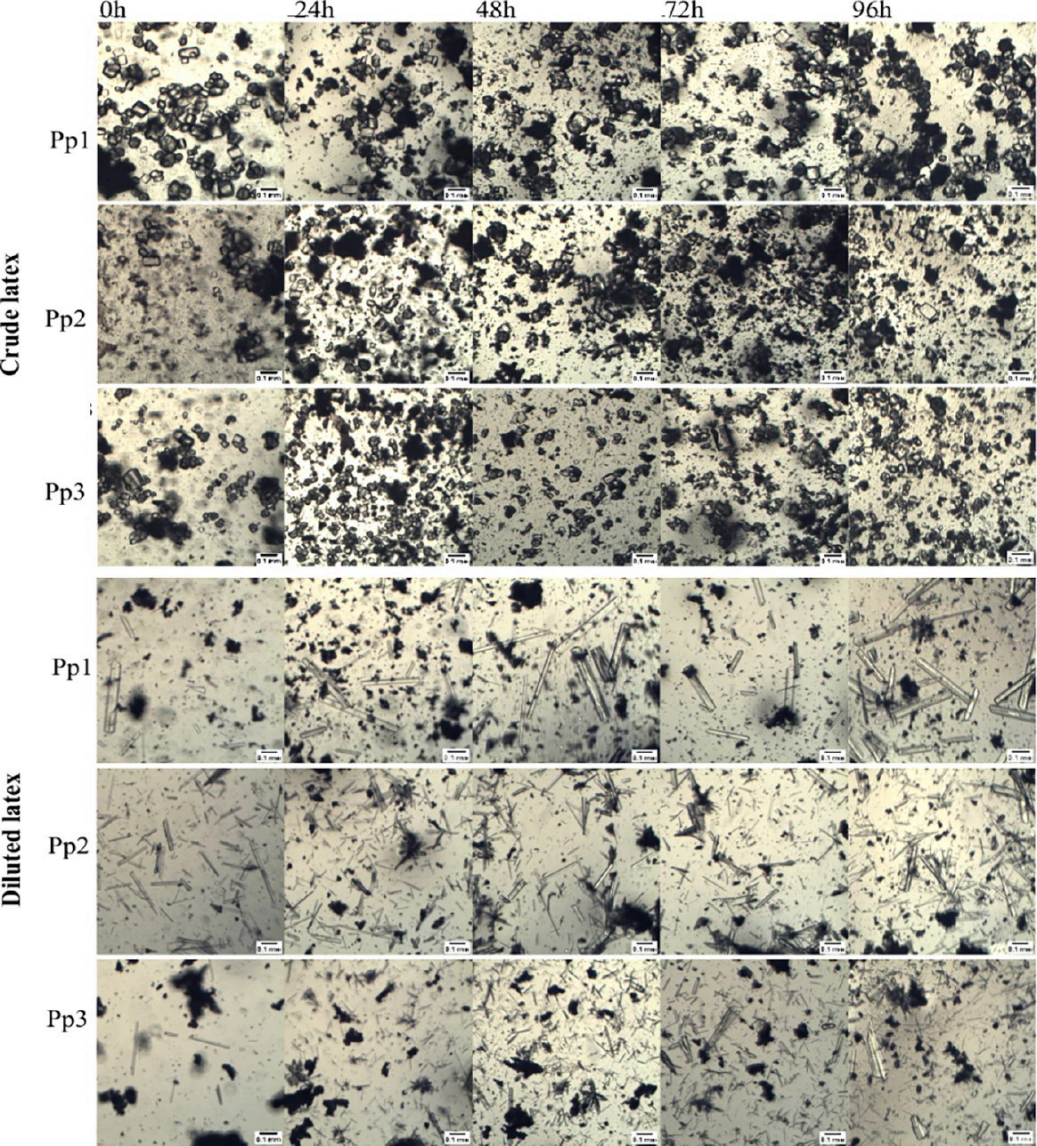
Crystal morphology observed in the latex of *Plumeria pudica* over a 96 h period at 25 °C. Images of crude latex and latex diluted in a 1:3 ratio with distilled water were obtained by using BEL Capture 3.2 software. Pp1, Pp2, and Pp3 denote the three independent samples of the *P. pudica* latex.

**Figure 3:**
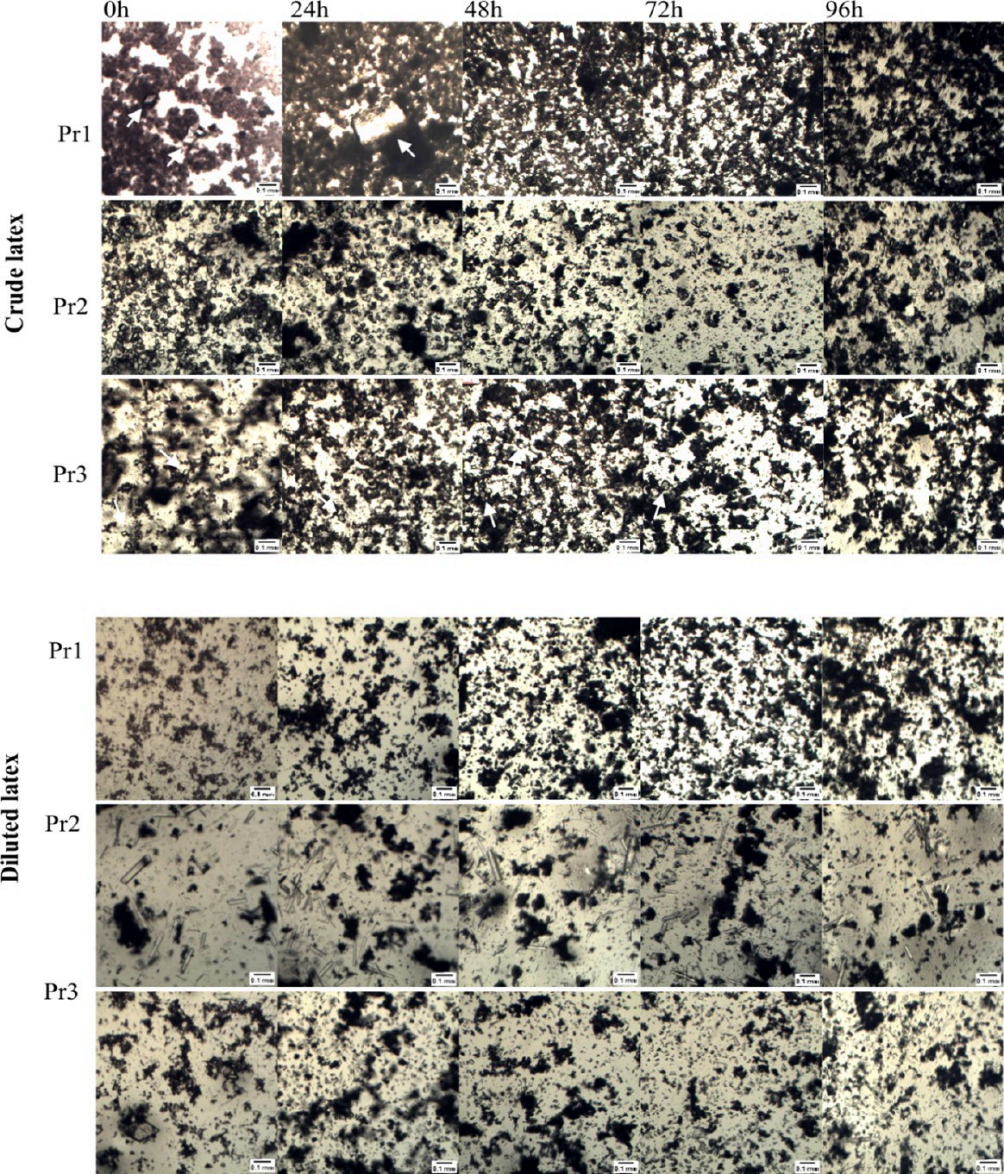
Crystal morphology observed in the latex of *Plumeria rubra* over a 96 h period at 25 °C. Images of a crude latex and a latex diluted in a 1:3 ratio with distilled water were obtained using BEL Capture 3.2 software. Pr1, Pr2, and Pr3 denote the three independent samples of the *P. rubra* latex. White arrows have been included in some images to indicate difficult-to-see crystals.

Single-crystal X-ray diffraction was utilized to determine the structures of the crystals. The raphide, styloid, crystalline sand, and prismatic crystals found in the latex of *P. pudica* consist of hydrated calcium malonate, represented by the following molecular formula: [Ca­(C_3_H_2_O_4_)­(H_2_O)_2_]·2H_2_O (Figure 4A). The crystalline structure of this compound was previously determined^18^ and documented in the Cambridge Structural Database (CSD). The present study is the first identification of calcium malonate crystals in plants, particularly within latexes. In the *P. rubra* latex, both raphides and styloid crystals are also composed of calcium malonate (Figure 4A).

**Figure 4:**
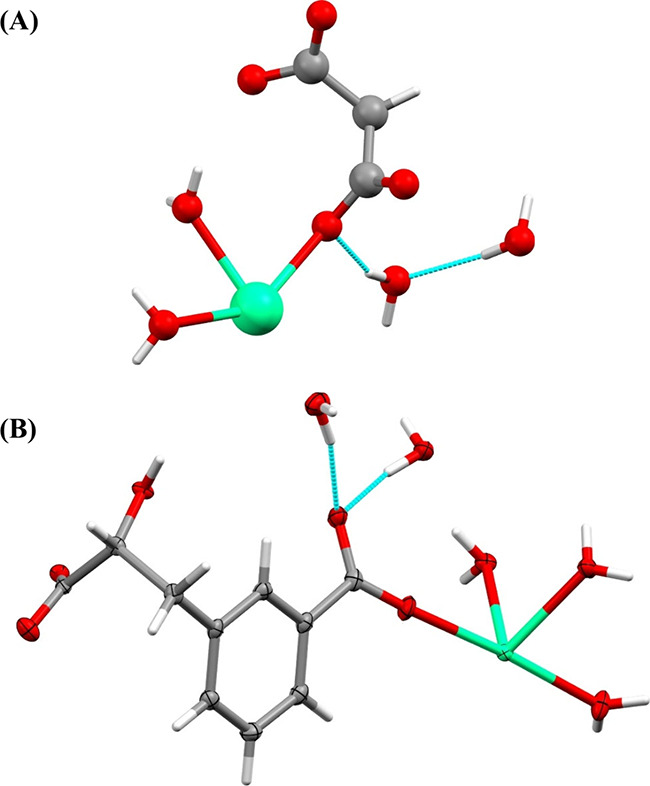
Crystal structures of the organic calcium salts identified in the latex of *Plumeria pudica* and *P. rubra*. (A) The raphides, styloid, crystalline sand, and prismatic crystals present in the latex of *P. pudica* along with the raphides and styloid crystals of *P. rubra* are composed of hydrated calcium malonate (CCDC: 1185166, no thermal ellipsoid data are given in this CSD entry). (B) Ortep plot (thermal ellipsoids at the 50% probability level) of the crystalline sand and prismatic crystals from the latex of *P. rubra*, composed of hydrated calcium cerberic acid B (CSD: 2269012). Calcium is depicted in green, carbon is depicted in gray, oxygen is depicted in red, and hydrogen is depicted in white. Figures were generated by utilizing the Mercury 4.2.0 software.

Nonetheless, crystalline sand and prismatic crystals consist of cerberic acid B. Cerberic acid B has been previously isolated from the bark of *Cerbera manghas* ^19^ and identified in dried flowers and the latex of *P. rubra*;^20,21^ however, this study is the first report of this component in the form of calcium crystals (C_20_H_34_Ca_2_O_19_) (Figure 4B). Both calcium salts, comprising malonic and cerberic acids B, yield polymeric structures. These structures have alternating calcium and organic molecules interconnected via carboxylic moieties. Typically, hydration water molecules are laterally associated with these polymeric structures, enhancing their stability through a network of hydrogen bonds. In the instance of cerberic acid B, it is noteworthy that the two hydration water molecules are also linked to the aromatic rings via OH−π interactions (Figure 4B).

The data related to the newly found hydrated calcium cerberic acid B salt crystalline structure can be accessed via the Cambridge Crystallographic Data Centre (accession number: 2269012). Table 1 and Tables S1–S3 display the data for this structure.

**Table 1:** Data of Hydrated Calcium Cerberic Acid B Crystals of the *Plumeria rubra* Latex^a^

CSD number	2269012
Formula	C_20_H_34_Ca_2_O_19_
Molar mass (g mol^–1^)	658.63
Temperature (K)	100.00
System	Orthorhombic
Space group	*P*2_1_2_1_2(18)
*a* (Å)	9.6027(2)
*b* (Å)	16.6731(4)
*c* (Å)	8.7673(2)
α (°)	90
β (°)	90
γ (°)	90
Volume (Å^3^)	1403.70(5)
Z	2
ρ_calc_ (g/cm^3^)	1.558
μ (mm^–1^)	4.302
F(000)	692
Crystal size (mm^3^)	0.2 × 0.15 × 0.026
Radiation	Cu*K*α (λ = 1.54178 Å)
Crystal color	Colorless
Crystal shape	Plate
2θ range (°)	10.09 – 144.35 (0.81 Å)
Index ranges	–10 ≤ *h* ≤ 11; −16 ≤ *k* ≤ 20; −10 ≤ *l* ≤ 10
Data collected	10306
Independent reflections	2647
	*R*_int_ = 0.0408
	*R*_sigma_ = 0.0414
Completeness to θ = 67.679°	99.4%
Data/constraints/parameters	2647/2/243
Goodness of fit on F^2^	1.051
Final R1 indices [I ≥ 2σ (I)]	*R*_1_ = 0.0299
	w*R*_2_ = 0.0745
Largest peak/hole [eÅ^–3^]	–0.29/0.34
Flack X Parameter	0.122(13)

a CSD: Cambridge Structural Database. Z: number of molecules in a unit cell. ρ: density. F(000): sum of the spreading factors in θ = 0. R1: concordance between calculated and observed models. I: intensity.

Despite the abundance of malonate in some plants, its function remains unclear. Some studies indicate that this compound plays a crucial role in nitrogen-fixing symbiosis,^22,23^ while others propose its involvement in carbon metabolism and energy production as it acts as a competitive inhibitor of succinate dehydrogenase enzyme.^24^ Its presence in mitochondria blocks the citric acid cycle, thereby inhibiting cellular respiration. Therefore, the efficient compartmentalization of this acid would prevent its strong inhibitory effect on metabolism.^25^

The formation of calcium malonate crystals is an uncommon occurrence, indicating an intriguing role for this compound in plant physiology. Besides carbon and energy metabolism regulation, the presence of calcium malonate crystals also suggests a mechanism for intracellular calcium regulation. Calcium is crucial for signaling and structural functions; however, it can become toxic when its levels surpass specific thresholds.^26^ The binding of calcium to malonate may serve as a dynamic reservoir, sequestering surplus calcium and reducing its potential harmful effects, particularly in scenarios of elevated calcium influx or oxidative stress.^27,28^ This sequestration may facilitate the regulated release of calcium during signaling events, including those induced by abiotic stresses or hormonal regulation.^29,30^ Therefore, the presence of calcium malonate in plant tissues may signify an advanced mechanism for regulating energy production, cellular stress, and various metabolic requirements. Moreover, calcium crystals can serve as a mechanism employed by plants for the detoxification of metals.^10^ These crystals may incorporate metals including aluminum, lead, cadmium, and strontium into their structure, thereby mitigating the potential toxic effects of these elements on plant metabolism.^31^ Accordingly, an alternative hypothesis posits that calcium malonate crystals may serve as a mechanism for metal detoxification in plants, as strontium was detected in the latex of *P. pudica* using an X-ray fluorescence spectrometer (data not published).

Although cerberic acid B has been detected in the bark, flowers, and latexes of some species,^19–21^ its physiological function remains largely underexplored. Salomé-Abarca and colleagues^21^ demonstrated that cerberic acid B is linked to antiherbivory activity of the latex of *P. rubra* against *Frankliniella occidentalis*. They also indicated that the activity could not be attributed only to cerberic acid B, as there was a decrease in antiherbivory activity following latex fractionation. In another study, *P. rubra* latex exhibited deterrent activity against oviposition by both *Callosobruchus maculatus* and *Zabrotis subfasciatus* beetles.^32^ This deterrent effect was abolished when the latex was fractionated into protein fractions, rubber, and small metabolites. These findings indicate that cerberic acid B in *P. rubra* latex may exhibit deterrent activity against insect oviposition through a repellent mechanism.

## Conclusions

Laticifers have distinct biochemical properties, regardless of their source or structural classification. Secondary metabolites and proteins are the most studied compounds. Here, crystal structures were identified only in *P. rubra* and *P. pudica*. A variety of sizes and shapes, including raphides, styloids, crystalline sands, and prismatic forms, were observed. All crystal forms associated with *P. pudica* were characterized as hydrated calcium malonate, while calcium malonate crystals were identified in the latex of *P. rubra* primarily as raphides and styloids. On the other hand, the crystalline sands and prismatic structures seen in the *P. rubra* latex were composed of cerberic acid B. The results show, for the first time, the presence of organic crystals in latex fluids, paving the way for further investigation into their functional significance. Some questions warrant elucidation: Are crystals inherently formed and stored in latex, or are they generated immediately following tissue damage? Are they triggered by herbivory or unfavorable environmental conditions? Due to the pivotal roles of calcium and malonate in different metabolic processes, additional investigation into the synthesis, distribution, and functional importance of calcium malonate in latex is necessary. Such investigations may uncover new systems of metabolic regulation and stress adaptation, offering insights that could ultimately refine agricultural methods and bolster plant resilience to fluctuating environmental conditions.

## Materials and Methods

### Plants and Latex Harvesting

This study examines the latex fluids obtained from four species belonging to the Apocynaceae family: *H. drasticus*, *C. procera*, *P. rubra*, and *P. pudica*. The fresh crude latex was collected from three distinct specimens of each species and analyzed separately. This investigation was properly registered in compliance with the prevailing Brazilian legislation within the SisGen system (https://sisgen.gov.br/paginas/login.aspx) under the code A689147. Latex fluids were systematically collected in the morning, specifically between 7:00 and 8:00 am, from healthy plants grown in the city of Fortaleza, Ceará, Brazil. A botanist identified all species, generating and storing exsiccates, at the Prisco Bezerra Herbarium at the Federal University of Ceará, Brazil.

The latex of *H. drasticus* was obtained from the trunk, while the latexes of the other three species were collected by cutting off the leaves.^16,33,34^ Three independent samples of crude latex (2 mL) were collected, sealed in Eppendorf tubes, maintained at 25 °C, and examined by using inverted light microscopy. Alternatively, the samples (0.5 mL of latex) were collected over a volume of water (1.5 mL) to reach a ratio of 1:3 (v:v). The latex samples of the three shrubs from *P. pudica* were named Pp1, Pp2, and Pp3, and those from three trees of *P. rubra* were named Pr1, Pr2, and Pr3.

### Light Microcopy Analysis

All samples were examined using an inverted light microscope at 10× magnification, 0, 24, 48, 72, and 96 h postcollection. For each analysis, a new aliquot was obtained from the corresponding tubes, which were maintained at 25 °C. The crystals were observed by adding 30 μL of each previously homogenized latex to slides, which were then covered with coverslips. The images were captured, and the crystal sizes were estimated using BEL Capture 3.2 and ImageJ software.

### X-ray Diffraction Analyses

Single-crystal X-ray diffraction data (ϕ scans and ω scans with κ and θ offsets) were collected on a Bruker D8 Venture κ-geometry diffractometer equipped with a Photon II CPAD detector and an IμS 3.0 Incoatec Cu Kα (λ = 1.54178 Å) microfocus source. For this, new samples of *P. pudica* and *P. rubra* latex were collected in distilled water (1:3 v/v), and all crystal forms found in each latex were selected and mounted on a Kapton fiber with a MiTeGen MicroMount using immersion oil. Data collections were performed at 100 K using an Oxford Cryostream cryostat (800 series Cryostream Plus) attached to the diffractometer. The APEX 4 software was used for the unit cell determination and data collection (Bruker AXS Inc., 2021). The data reduction and global cell refinement were carried out using the Bruker SAINT+ software package (Bruker AXS Inc., 2019), and a numerical absorption correction was performed with SADABS.^35^ Using the Olex2 ^36^ interface program to the SHELX suite, the structure was solved by the intrinsic phasing method implemented in ShelXT,^37^ allowing the location of most of the non-hydrogen atoms. The remaining non-hydrogen atoms were located from different Fourier maps calculated from successive full-matrix least-squares refinement cycles on F2 with ShelXL^38^ and refined using anisotropic displacement parameters. Hydrogen atoms were placed according to geometrical criteria and treated using the riding model. MERCURY version 4.2.0 was used to create images. Detailed structural data are accessed in the Supporting Information found in Tables S1–S3.

## Supplementary Material



## Notes

Tables S1–S3 present atomic coordinates and Ueq [Å2], bond lengths and angles, and torsion angles of hydrated calcium cerberic acid B crystals from the *Plumeria rubra* latex (PDF)
