# Supplementary material for: Identification and Characterization of Organic Calcium Crystals in the Plumeria pudica and Plumeria rubra Latexes
Source: ACS Omega. 2025 Jul 11;10(28):30879–85. doi: 10.1021/acsomega.5c03229 (PMC12290934; doi:10.1021/acsomega.5c03229)
Supplement: Supplementary file 1 [file ao5c03229_si_001.pdf]

Identification and characterization of organic calcium crystals in the *Plumeria pudica* and *P. rubra* latex

Larissa Barbosa Nogueira Freitas[a], Cleverson Diniz Teixeira de Freitas[a*], Alejandro Pedro Ayala[b], Thiago Alves de Moura[c], Jefferson Soares de Oliveira[d], José Francisco de Carvalho Gonçalves[e], Márcio Viana Ramos[a*]

[a]Department of Biochemistry and Molecular Biology, Federal University of Ceará, Fortaleza, Ceará, Brazil.

[b]Department of Physics, Federal University of Ceará, Fortaleza, Ceará, Brazil

[c]Federal Institute of Education, Science and Technology of Ceará, Acaraú, Ceará.

[d]Department of Biomedicine, Federal University of the Parnaíba Delta, Parnaíba, Piauí, Brazil.

[e]Plant Physiology and Biochemistry Laboratory, National Institute of Amazonian Research - MCTI-INPA, Manaus, Brazil.

*Corresponding authors: Cleverson Diniz Teixeira de Freitas (cleversondiniz@ufc.br) and Márcio Viana Ramos (vramos@ufc.br). Department of Biochemistry and Molecular Biology, Federal University of Ceará, Fortaleza, Ceará, Brazil.

Table S1. Atomic coordinates and Ueq [Å^2] of 2269012 (number CCDC).

**Atom**	***x***	***y***	***z***	***U*eq**
Ca1	0.31009(5)	0.54384(3)	−0.00618(6)	0.00972(15)
O4	0.55391(19)	0.58186(10)	0.9936(2)	0.0116(4)
O5	0.3493(2)	0.68120(12)	0.9334(2)	0.0133(4)
O2	0.2114(2)	0.57962(12)	0.4732(2)	0.0176(4)
O5W	0.000000	0.500000	0.3288(4)	0.0188(6)
O3W	0.1418(2)	0.44769(13)	0.0807(3)	0.0143(4)
O3	0.7037(2)	0.68100(11)	1.0429(2)	0.0173(4)
O2W	0.0942(2)	0.59878(13)	−0.0806(3)	0.0178(5)
O1	0.3192(3)	0.57883(13)	0.2481(2)	0.0193(4)
O1W	0.3175(3)	0.50443(13)	−0.2654(2)	0.0220(5)
H1WA	0.246975	0.525413	−0.314131	0.033
H1WB	0.304100	0.452776	−0.272221	0.033
O4W	0.5393(3)	0.82475(15)	0.3490(3)	0.0262(5)
H4WA	0.589446	0.848483	0.418226	0.039
H4WB	0.550726	0.773817	0.366602	0.039
C7	0.5882(3)	0.65504(15)	0.9998(3)	0.0117(5)
C2	0.4436(3)	0.62796(16)	0.4620(3)	0.0140(6)
C3	0.4299(3)	0.67513(17)	0.5941(3)	0.0139(6)
C5	0.5293(3)	0.76112(17)	0.8042(3)	0.0135(6)
C4	0.5469(3)	0.70686(17)	0.6674(3)	0.0137(6)
C6	0.4821(3)	0.71839(16)	0.9513(3)	0.0116(6)
C1	0.5765(3)	0.61270(17)	0.4036(3)	0.0162(6)
C9	0.6932(3)	0.64212(16)	0.4780(3)	0.0171(6)
C8	0.6789(3)	0.68904(17)	0.6090(3)	0.0160(6)
C10	0.3153(3)	0.59326(16)	0.3876(3)	0.0153(6)
H3	0.338(4)	0.685(2)	0.632(4)	0.012(8)
H5A	0.612(4)	0.790(2)	0.824(4)	0.006(7)
H9	0.784(4)	0.630(2)	0.439(4)	0.011(8)
H5B	0.450(4)	0.803(2)	0.784(4)	0.012(8)
H6	0.477(3)	0.7569(19)	1.036(4)	0.004(7)
H5	0.290(5)	0.714(3)	0.936(5)	0.034(12)
H8	0.760(4)	0.707(2)	0.659(4)	0.010(8)
H1	0.582(4)	0.585(3)	0.309(5)	0.023(10)
H2WA	0.078(4)	0.621(3)	−0.165(6)	0.026(11)
H2WB	0.021(6)	0.585(3)	−0.047(6)	0.042(14)
H3WA	0.187(5)	0.406(3)	0.084(5)	0.035(12)
H3WB	0.107(5)	0.457(3)	0.152(6)	0.034(13)
H5W	0.080(5)	0.537(4)	0.348(7)	0.10(2)

U_{eq} is defined as 1/3 of the trace of the orthogonalized U_{ij} tensor.

Table S2. Bond lengths and angles of 2269012 (Number CCDC).

**Atom-Atom**	Lenght (Å)
Ca1–O4$^{\#1}$	2.4256(19)
Ca1–O5$^{\#1}$	2.381(2)
Ca1–O3W	2.401(2)
Ca1–O2W	2.359(2)
Ca1–O1	2.306(2)
Ca1–O1W	2.367(2)
Ca1–H1WA	2.7841
O3W–H3WB	0.73(5)
O3–C7	1.249(3)
O2W–H2WA	0.84(5)
O2W–H2WB	0.80(6)
O1–C10	1.247(4)
O1W–H1WA	0.8735
O1W–H1WB	0.8729
O4W–H4WA	0.8699
O4W–H4WB	0.8700
C7–C6	1.528(4)
C2–C3	1.406(4)
C2–C1	1.398(4)
C2–C10	1.510(4)
C3–C4	1.399(4)
C3–H3	0.96(4)
C5–C4	1.512(4)
C5–C6	1.542(4)
C5–H5A	0.94(4)
C5–H5B	1.05(4)
C4–C8	1.399(4)
C6–H6	0.98(3)
C1–C9	1.386(4)
C1–H1	0.95(4)
C9–C8	1.397(4)
C9–H9	0.96(4)
C8–H8	0.94(4)
**Atom-Atom-Atom**	**Angle (°)**
O4$^{\#1}$–Ca1–H1WA	103.8
O4$^{\#1}$–Ca1–H1WB	99.3
O4$^{\#1}$–Ca1–H3WA	130.1(11)
O5$^{\#1}$–Ca1–O4$^{\#1}$	66.17(7)
O5$^{\#1}$–Ca1–O3W	145.03(8)
O5$^{\#1}$–Ca1–H1WA	85.7
O5$^{\#1}$–Ca1–H1WB	110.0
O5$^{\#1}$–Ca1–H3WA	162.3(11)
O3W–Ca1–O4$^{\#1}$	145.56(7)
O3W–Ca1–H1WA	95.0
O3W–Ca1–H1WB	83.5
O3W–Ca1–H3WA	17.3(11)
O2W–Ca1–O4$^{\#1}$	138.31(8)

O2W–Ca1–O5$^{\#1}$	72.76(8)
O2W–Ca1–O3W	75.83(8)
O2W–Ca1–O1W	82.44(9)
O2W–Ca1–H1WA	65.4
O2W–Ca1–H1WB	87.8
O2W–Ca1–H3WA	91.5(11)
O1–Ca1–O4$^{\#1}$	84.13(8)
O1–Ca1–O5$^{\#1}$	88.03(7)
O1–Ca1–O3W	83.57(8)
O1–Ca1–O2W	101.69(9)
O1–Ca1–O1W	175.84(9)
O1–Ca1–H1WA	166.8
O1–Ca1–H1WB	161.6
O1–Ca1–H3WA	87.2(10)
O1W–Ca1–O4$^{\#1}$	92.47(8)
O1W–Ca1–O5$^{\#1}$	92.79(7)
O1W–Ca1–O3W	98.01(8)
O1W–Ca1–H1WA	17.2
O1W–Ca1–H1WB	17.2
O1W–Ca1–H3WA	93.2(10)
H1WA–Ca1–H1WB	28.7
H1WA–Ca1–H3WA	95.3
H1WB–Ca1–H3WA	76.7
Ca1$^{\#2}$–O4–Ca1$^{\#3}$	106.78(7)
C7–O4–Ca1$^{\#2}$	120.22(16)
C7–O4–Ca1$^{\#3}$	132.92(16)
Ca1$^{\#2}$–O5–H5	123(3)
C6–O5–Ca1$^{\#2}$	122.32(16)
C6–O5–H5	110(3)
Ca1–O3W–H3WA	103(3)
Ca1–O3W–H3WB	116(4)
H3WA–O3W–H3WB	113(5)
Ca1–O2W–H2WA	125(3)
Ca1–O2W–H2WB	124(4)
H2WA–O2W–H2WB	107(5)
C10–O1–Ca1	174.7(2)
Ca1–O1W–H1WA	109.6
Ca1–O1W–H1WB	109.6
H1WA–O1W–H1WB	104.3
H4WA–O4W–H4WB	104.5
O4–C7–C6	118.8(2)
O3–C7–O4	125.3(2)
O3–C7–C6	115.9(2)
C3–C2–C10	119.6(3)
C1–C2–C3	119.3(3)
C1–C2–C10	121.1(2)
C2–C3–H3	118(2)
C4–C3–C2	121.0(3)
C4–C3–H3	121(2)
C4–C5–C6	114.8(2)

C4–C5–H5A	111(2)
C4–C5–H5B	110.3(19)
C6–C5–H5A	109(2)
C6–C5–H5B	103.6(19)
H5A–C5–H5B	108(3)
C3–C4–C5	120.1(3)
C8–C4–C3	118.6(3)
C8–C4–C5	121.3(3)
O5–C6–C7	109.1(2)
O5–C6–C5	111.8(2)
O5–C6–H6	109(2)
C7–C6–C5	110.8(2)
C7–C6–H6	106.0(19)
C5–C6–H6	110.1(19)
C2–C1–H1	117(3)
C9–C1–C2	120.1(3)
C9–C1–H1	123(3)
C1–C9–C8	120.4(3)
C1–C9–H9	119(2)
C8–C9–H9	121(2)
C4–C8–H8	121(2)
C9–C8–C4	120.6(3)
C9–C8–H8	119(2)
O2–C10–C2	117.0(2)
O1–C10–O2	124.7(3)
O1–C10–C2	118.3(3)

Symmetry transformations used to generate equivalent atoms: #1: +X, +Y, -1+Z; #2: +X, +Y, 1+Z; #3: 1-X, 1-Y, 1+Z

Table S3. Torsion angles of 2269012 (Number CCDC).

Atom-Atom-Atom-Atom	Torsion angle (°)
$Ca1^{\#1}$–O4–C7–O3	158.6(2)
$Ca1^{\#2}$–O4–C7–O3	−17.6(5)
$Ca1^{\#1}$–O4–C7–C6	−21.4(3)
$Ca1^{\#2}$–O4–C7–C6	162.39(18)
$Ca1^{\#1}$–O5–C6–C7	4.6(3)
$Ca1^{\#1}$–O5–C6–C5	127.6(2)
O4–C7–C6–O5	10.9(4)
O4–C7–C6–C5	−112.6(3)
O3–C7–C6–O5	−169.1(2)
O3–C7–C6–C5	67.4(3)
C2–C3–C4–C5	176.7(3)
C2–C3–C4–C8	−1.6(4)
C2–C1–C9–C8	−1.8(4)
C3–C2–C1–C9	1.7(4)
C3–C2–C10–O2	−27.8(4)
C3–C2–C10–O1	153.4(3)
C3–C4–C8–C9	1.6(4)
C5–C4–C8–C9	−176.7(3)
C4–C5–C6–O5	−61.4(3)
C4–C5–C6–C7	60.5(3)
C6–C5–C4–C3	72.4(3)
C6–C5–C4–C8	−109.3(3)
C1–C2–C3–C4	0.0(4)
C1–C2–C10–O2	150.9(3)
C1–C2–C10–O1	−27.9(4)
C1–C9–C8–C4	0.2(4)
C10–C2–C3–C4	178.8(3)
C10–C2–C1–C9	−177.0(3)

Symmetry transformations used to generate equivalent atoms:#1: +X, +Y, 1+Z; #2: 1-X, 1-Y, 1+Z;
